# Aqueous Humor Outflow Requires Active Cellular Metabolism in Mice

**DOI:** 10.1167/iovs.61.10.45

**Published:** 2020-08-26

**Authors:** Ester Reina-Torres, Alexandra Boussommier-Calleja, Joseph M. Sherwood, Darryl R. Overby

**Affiliations:** 1 Department of Bioengineering, Imperial College London, London, United Kingdom; 2 ImVitro, Paris, France

**Keywords:** aqueous flow, outflow resistance, metabolism, animal models, temperature

## Abstract

**Purpose:**

Conventional wisdom posits that aqueous humor leaves the eye by passive bulk flow without involving energy-dependent processes. However, recent studies have shown that active processes, such as cell contractility, contribute to outflow regulation. Here, we examine whether inhibiting cellular metabolism affects outflow facility in mice.

**Methods:**

We measured outflow facility in paired enucleated eyes from C57BL/6J mice using iPerfusion. We had three Experimental Sets: ES1, perfused at 35°C versus 22°C; ES2, perfused with metabolic inhibitors versus vehicle at 35°C; and ES3, perfused at 35°C versus 22°C in the presence of metabolic inhibitors. Inhibitors targeted glycolysis and oxidative phosphorylation (2-deoxy-D-glucose, 3PO and sodium azide). We also measured adenosine triphosphate (ATP) levels in separate murine anterior segments treated like ES1 and ES2.

**Results:**

Reducing temperature decreased facility by 63% [38%, 78%] (mean [95% confidence interval (CI)], *n* = 10 pairs; *P* = 0.002) in ES1 after correcting for changes in viscosity. Metabolic inhibitors reduced facility by 21% [9%, 31%] (*n* = 9, *P* = 0.006) in ES2. In the presence of inhibitors, temperature reduction decreased facility by 44% [29%, 56%] (*n* = 8, *P* < 0.001) in ES3. Metabolic inhibitors reduced anterior segment adenosine triphosphate (ATP) levels by 90% [83%, 97%] (*n* = 5, *P*<<0.001), but reducing temperature did not affect ATP.

**Conclusions:**

Inhibiting cellular metabolism decreases outflow facility within minutes. This implies that outflow is not entirely passive, but depends partly on energy-dependent cellular processes, at least in mice. This study also suggests that there is a yet unidentified mechanism, which is strongly temperature-dependent but metabolism-independent, that is necessary for nearly half of normal outflow function in mice.

Aqueous humor drainage through the trabecular meshwork (TM) and into Schlemm's canal (SC) is thought to occur by passive bulk flow without active transport or energy-dependent cellular processes. Contributing to this view, Bárány^1^ found that outflow facility was unaffected by several metabolic inhibitors in enucleated cattle eyes. VanBuskirk and Grant^2^ found that outflow facility was insensitive to temperature change in enucleated human eyes. These classic studies suggest that TM or SC cells do not actively regulate outflow or contribute directly to outflow function. This led naturally to the view that the outflow pathway functions like a passive filter, without a direct role for the TM or SC cells themselves.^3^^–^^11^

The notion that outflow functions like a passive filter is incongruent with recent findings showing that metabolically-dependent cellular processes, such as cell contractility,^12^^–^^17^ exocytosis,^18^^,^^19^ and signal transduction,^20^^–^^24^ affect conventional outflow facility. There are also two studies that, in contrast to VanBuskirk and Grant,^2^ suggest that outflow facility is temperature dependent in enucleated mouse^25^ and calf eyes.^26^ Thus it remains an open question as to what extent aqueous humor outflow requires active cellular metabolism. The goal of this study was to assess the effects of reduced temperature and metabolic inhibitors on outflow facility in freshly enucleated mouse eyes.

## Materials and Methods

### Experimental Design

In this project, we examined the role of active cellular metabolism on outflow facility in ex vivo mouse eyes. We inhibited metabolic activity by reducing temperature or by perfusing with chemical inhibitors of cellular metabolism. We had three experimental sets. In Experimental Set 1 (ES1), we examined the effect of temperature reduction by perfusing paired eyes at 22°C versus 35°C, with both eyes receiving standard perfusate. In Experimental Set 2 (ES2), we perfused paired eyes with or without metabolic inhibitors, with both eyes at 35°C. In Experimental Set 3 (ES3), we examined the effect of temperature independently of metabolism by perfusing paired eyes at 22°C versus 35°C, with both eyes receiving metabolic inhibitors. We also tested how temperature reduction and metabolic inhibitors affect intracellular ATP levels in the anterior segment.

To inhibit cellular metabolism, we used a combination of drugs that target two pathways involved in ATP production, glycolysis and oxidative phosphorylation. To inhibit glycolysis, we used 2-deoxy-D-glucose (2-DG) and (2E)-3-(3-pyridinyl)-1-(4-pyridinyl)-2-propen-1-one (3PO). The 2-DG is a glucose analogue that is internalized by cells where it competitively inhibits the production of glucose-6-phosphate to prevent further glycolysis.^27^^,^^28^ The 3PO inhibits 6-phosphofructo-2-kinase/fructose-2,6-biphosphatase 3 (PFKFB3),^29^^,^^30^ which is a rate-limiting enzyme in glycolysis. To inhibit oxidative phosphorylation, we used sodium azide (NaN_3_) that blocks electron transport in mitochondria.^31^^,^^32^

### Animal Handling

This study used 40 male C57BL/6J mice (Charles River Laboratory, UK) aged between 10 and 13 weeks. Mice were fed ad libitum and kept in a 12-hour light/dark cycle. All mice were handled in accordance with the ARVO Statement for the Use of Animals in Ophthalmic and Vision Research under the authority of a UK Home Office Project License. Mice were humanely culled by cervical dislocation, and death was confirmed by scission of the femoral vein. Eyes were surgically enucleated within 10 minutes of death by cutting the extraocular muscles and the optic nerve with fine curved scissors. Once enucleated, eyes were stored in phosphate-buffered saline solution (PBS) at room temperature until perfusion (less than 20 minutes). All eyes were perfused within 30 minutes postmortem.

### Measurement of Outflow Facility

We measured outflow facility using iPerfusion.^33^ Briefly, iPerfusion consists of a thermal flow sensor (SLG150; Sensirion, Staefa, Switzerland) and a differential pressure transducer (PX409; Omegadyne, Sunbury, OH, USA) to measure the flow rate *Q* into the eye and the pressure *P* within the eye. An actuated reservoir is used to control the upstream applied pressure. The temperature of the eye is maintained in a bath of PBS with a computer-controlled heater that maintains the bath at the desired temperature, either 35° ± 0.5°C or 22° ± 0.2°C.

Within 10 minutes of enucleation, the eye was adhered to a support platform within the bath using tissue glue. The eye was then quickly submerged by filling the bath with PBS that was prewarmed to the desired temperature. We used a pulled glass micropipette to cannulate the eye via the anterior chamber for perfusion. The micropipette was beveled with a tip diameter of approximately 100 µm. Before cannulation, we measured the resistance of the micropipette, which would indicate whether there was a blockage or bubble that would require removal. The eye was then cannulated at an applied pressure of 8 mm Hg. Cannulation was done using a micromanipulator under a stereoscopic microscope. We measured outflow facility in two eyes simultaneously using two iPerfusion systems.

**Figure 1.**
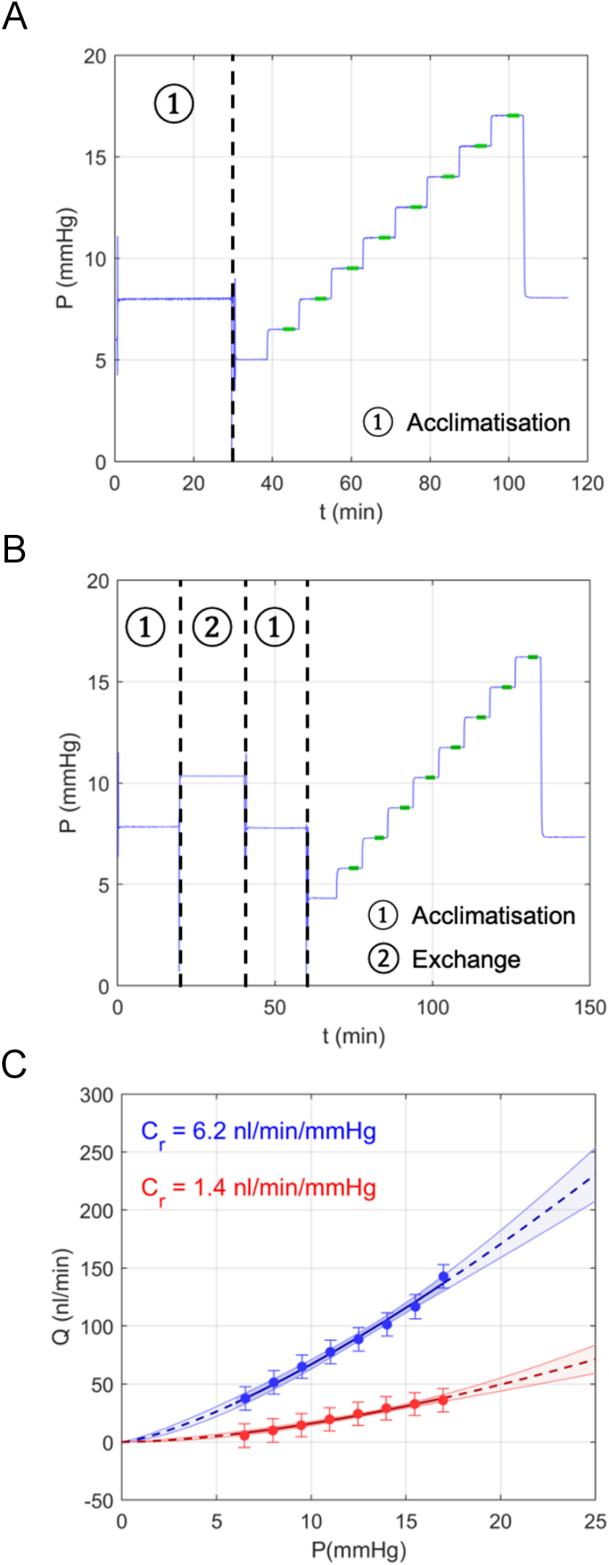
(**A)** The pressure stepping protocol used for Experimental Set (ES) 1 examining the effect of temperature. The green highlights show data used for the fitting by Equation 1. (**B)** The Pressure stepping protocol used for ES2 and ES3 that included an anterior chamber exchange to deliver metabolic inhibitors. (**C)** Flow (*Q*) and pressure (*P*) data for a pair of eyes from ES1 perfused at 22°C (*red*) or 35°C (*blue*). Data at 22°C are not viscosity corrected. Error bars represent 95% CI on each measurement. The curves represent the best fit by Equation 1, with *C_r_* values shown for each case. The shading around the curve depicts the 95% CI on the fit.

A standard perfusion consisted of an initial pressurization step of 30 minutes at 8 mm Hg for acclimatization, followed by nine discrete pressure steps from 5 to 18.5 mm Hg and a final step at 8 mm Hg (Fig. 1A). The duration of each step was typically eight to ten minutes and was controlled by the iPerfusion software based on a stability condition that advanced to the next step when *Q* changed by no more than 3 nL/min per minute over a six-minute moving window. Each step typically required two to four minutes to achieve stability.

Outflow facility was calculated as described by Sherwood et al*.*^33^ Briefly, *Q* and *P* were measured over a sequence of nine increasing pressure steps, and the data were fit by the following power-law relationship:

(1) $$Q=C_{r}{\left(\frac{P}{P_{r}}\right)}^{\beta }P$$

where *C_r_* is the reference value of outflow facility that applies at a reference pressure *P_r_*, defined to be 8 mm Hg to correspond to the physiological pressure drop across the outflow pathway (Fig. 1C). The βcaptures the nonlinearity of the *Q*-*P* relationship, which is perfectly linear when β = 0. Equation 1 requires that *Q* = 0 when *P* = 0, as has been confirmed in enucleated mouse eyes.^34^ The *Q* and *P* values used for the fit were taken as the average over the last 300 seconds of each step that achieves our stability criterion. Any steps not reaching our stability criterion were excluded from the fit, along with any subsequent steps. All perfusions had at least six steps, and only increasing pressure steps were included in the fit (i.e., those shown in green highlights in Fig. 1).

The effect of a specific treatment on outflow facility was determined by calculating the average change in *C_r_* between contralateral eyes. In this way, we account for the correlation in outflow facility between contralateral eyes,^33^ and variations in baseline outflow facility between individual mice do not unduly influence the assessment of the relative difference in facility in response to the treatment. Statistical significance on the relative difference in outflow facility was determined using a weighted paired *t*-test as described previously.^33^ Any change in *C_r_* that exceeded 2.5 times the median absolute deviation from the median change was considered an outlier.^33^

To examine the relationship between the relative difference in facility and the baseline outflow facility (defined as the value of ln*C_r_* in the control eye), we used linear regression analysis for ES2 or analysis of covariance (ANCOVA) for ES1 and ES3. To consider the combined effects of temperature reduction in ES1 and ES3, we defined treatment with metabolic inhibitors within the ANCOVA as a categorical variable.

### ES1: Investigating the Effects of Temperature Reduction

To investigate the effect of temperature reduction on outflow facility, one eye of a pair was perfused at a physiological temperature of 35°C while the contralateral eye was perfused at 22°C. Both eyes were perfused with Dulbecco's PBS containing divalent cations and 5.5 mmol/L glucose that was passed through a 0.22 µm filter before use. ES1 used 10 mice, which were perfused using the pressure-stepping protocol summarized in Figure 1A.

Measurements of outflow facility at different temperatures require correction to account for changes in perfusate viscosity. Assuming that the viscosity of the perfusate behaves like that of water, the value of *C_r_* measured at 22°C must be multiplied by the ratio $\mu_{22^{\circ }C}/\mu_{35^{\circ }C}$, where $\mu_{22^{\circ }C}$ is the dynamic viscosity of water at 22°C (954 Pa.s) and $\mu_{35^{\circ }C}$ is the dynamic viscosity of water at 35°C (719 Pa.s).^35^ Note that this ratio is greater than unity, meaning that the corrected facility is larger than the measured facility (i.e., the correction reduces, not increases, the apparent effect of temperature on facility). The corrected outflow facility represents the facility that would have been measured if perfusate viscosity were unaffected by temperature. Thus, by comparing the corrected *C_r_* at 22°C versus the measured *C_r_* at 35°C, we can isolate the effects of temperature on outflow facility, independent of changes in viscosity.

### ES2: Investigating the Effects of Metabolic Inhibitors

To test the effect of the metabolic inhibitors, paired eyes were perfused with or without metabolic inhibitors at a physiological temperature of 35°C. The inhibitors targeted glycolysis and oxidative phosphorylation and included 11 mmol/L 2-DG, 4 mmol/L NaN_3_, and 100 µmol/L 3PO (Calbiochem, Merck, Darmstadt, Germany) in Dulbecco's PBS without additional glucose. The contralateral eye not receiving metabolic inhibitors was perfused with isosmotic vehicle, which was Dulbecco's PBS containing 11 mmol/L glucose, 4 mmol/L NaCl and 0.2% DMSO. All solutions were filtered through a 0.22 µm filter before use. ES2 used 10 mice.

We used anterior chamber exchange to deliver a known concentration of metabolic inhibitors that is maintained throughout the perfusion. A sham exchange was performed for the eye receiving isosmotic vehicle. To perform an exchange, the eye was cannulated with two micropipettes held in a custom device mounted on a micromanipulator to allow for simultaneous dual cannulation. Both micropipette tips were positioned in the anterior chamber. One micropipette was connected to the iPerfusion system, whereas the other was connected to a 1 mL glass syringe (Gastight 1700 Series, Hamilton Company, Reno, NV, USA) mounted on a syringe pump (PHD ULTRA; Harvard Apparatus, Holliston, MA, USA). The micropipette and upstream tubing connecting to iPerfusion were filled with perfusion fluid containing metabolic inhibitors (or isosmotic vehicle). To perform the exchange, the syringe pump was programmed to withdraw fluid from the anterior chamber at a flow rate of 5 µL/min for 20 minutes. This draws fluid into the eye through the micropipette connected to iPerfusion, thereby filling the anterior chamber with the desired concentration of drug or vehicle. The eye pressure during the exchange was controlled using an actuated reservoir set to give an applied upstream pressure *P_a_* of:

(2) $$P_{a}=P_{e}+Q_{e}\;R_{n}$$

where *P_e_* is the desired value of IOP during the exchange (defined to be 8 mm Hg), *Q_e_* is the exchange flow rate (5 µL/min) and *R_n_* is the resistance of the micropipette (typical range of 0.1–1.0 mm Hg/µL/min). We measured *R_n_* before each experiment by placing the pipette tip in the eye bath and using iPerfusion to measure its resistance. To ensure a correct exchange, both micropipettes should have a similar resistance. Equation 2 accounts for the pressure drop across the micropipette, which is normally negligible when flow rates are small (< 200 nL/min), as occurs during a perfusion, but must be accounted for when flow rates are large, as occurs during the exchange.

ES2 used the following pressure stepping protocol. After cannulation, the eye was acclimated to the perfusion system for 20 minutes at an applied pressure of 8 mm Hg. The anterior chamber was then exchanged with either metabolic inhibitors or vehicle at 8 mm Hg for 20 minutes, as described above. The eye was then allowed to further acclimate after the exchange for 20 minutes at 8 mm Hg. The eye was then perfused over nine increasing pressure steps as for ES1 to determine *C_r_* (Fig. 1B).

### ES3: Investigating the Effect of Temperature, Independent of Metabolic Inhibitors

Temperature reduction examined in ES1 will tend to suppress enzyme activity, and thereby reduce metabolic processes. This should mimic the effects of the metabolic inhibitors examined in ES2. However, it is possible that temperature reduction has additional effects on outflow, for example by affecting the mechanical properties of cells, such as cell stiffness^36^ and contractility.^37^^,^^38^ We thus investigated the effects of temperature independent of metabolic inhibitors. To do this, we put metabolic inhibitors into both eyes, but perfused one eye at 35°C while the contralateral was perfused at 22°C. The cocktail of metabolic inhibitors was the same as that for ES2, delivered into both eyes using an anterior chamber exchange, as described above. Outflow facility at 22°C was corrected to account for changes in viscosity, as described for ES1. The pressure stepping protocol was identical to that used for ES2 (Fig. 1B). ES3 used 10 mice.

### Measurement of ATP in the Anterior Segment

Our aim was to assess how temperature reduction and the metabolic inhibitors affect ATP. Because of the difficulty of manually dissecting the tiny outflow pathway of mice, we resorted to measuring ATP levels in the anterior segment, which includes the cornea, corneoscleral rim, and conventional outflow pathway. These measurements used 10 mice.

The mice were handled as described above for perfusions. After enucleation, eyes were stored in PBS at room temperature and, within 20 minutes, eyes were hemisected posteriorly to the limbus. The posterior segment and lens were discarded, and the iris was carefully removed. This was necessary because the heavy iris pigmentation interfered with the luminescence measurements described below. Anterior segments were then incubated with metabolic inhibitors versus isosmotic controls at 35°C (*n* = 5 pairs) or incubated in standard perfusion solution at 35°C versus 22°C (*n* = 5 pairs) for two hours. These two conditions mimic what the eyes experience during perfusion for ES1 and ES2.

After incubation, the tissues were washed in ice-cold PBS and homogenized in 500 µL of cold lysis buffer (10 mmol/L Tris pH 7.5, 100 mmol/L NaCl, 1 mmol/L ethylenediamine tetra-acetic acid and 0.01% Triton X-100 in dH_2_O) using a tissue homogenizer (T10 basic Ultra-Turrax, IKA, Staufen, Germany). The homogenization was done in three cycles of 15 seconds leaving the tubes in ice for 10 seconds in between each cycle. Tissue homogenates were spun in a centrifuge at 17 × 10^3^*g* for five minutes at 4°C, and the supernatant was kept in ice for ATP and protein quantification. ATP levels were measured by luminescence using an ATP determination kit (A22066; ThermoFisher Scientific, Waltham, MA, USA) following manufacturer's instructions. To normalize for the quantity of tissue, protein concentration was measured using the Pierce BCA Protein Assay Kit (no. 23227, ThermoFisher Scientific) following manufacturer's instructions. We calculated the quantity of ATP (in picomoles) per unit mass of protein (in milligrams) and determined the relative change in normalized ATP levels between contralateral eyes. Statistical significance was evaluated using a *t*-test on the average percentage change in ATP between contralateral eyes.

## Results

### Effect of Temperature and Metabolic Inhibitors on Outflow Facility

**Figure 2.**
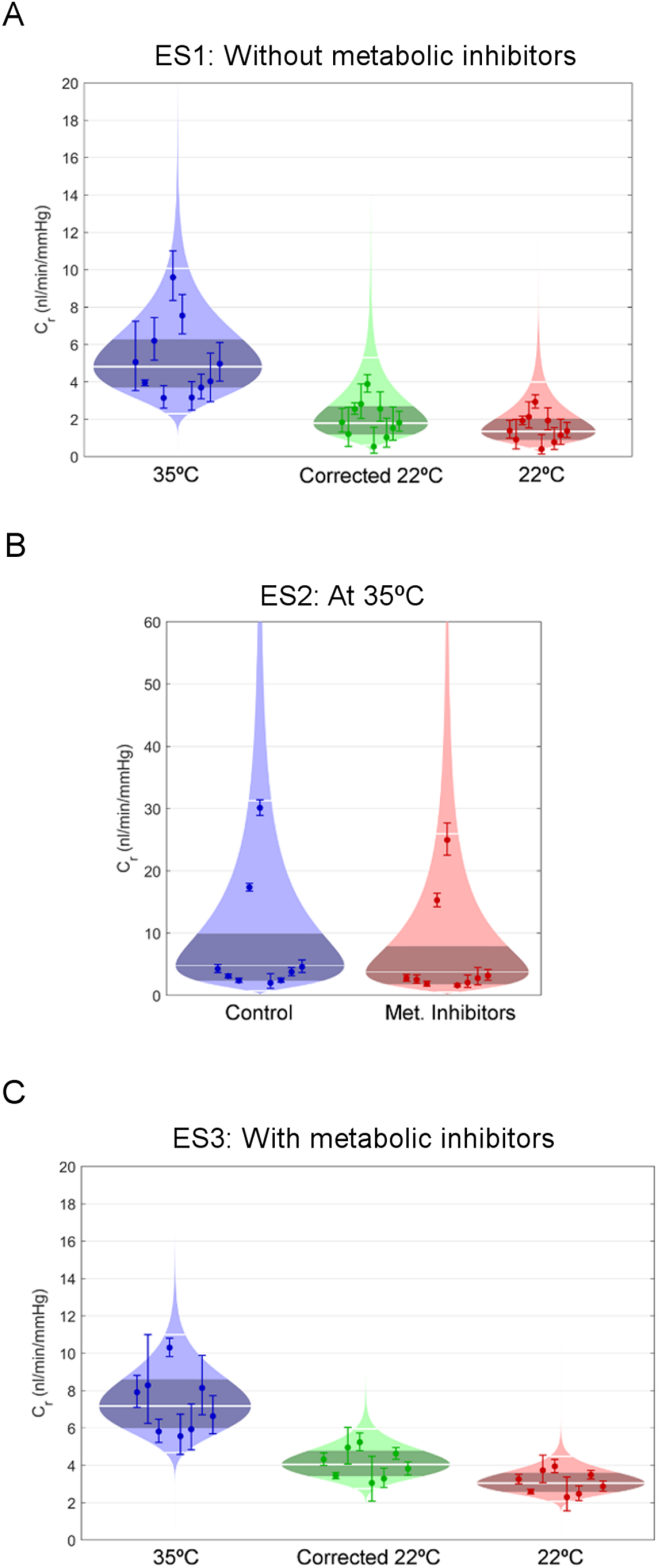
Cello plots of outflow facility (*C_r_*) data from ES1 (A), ES2 (B) and ES3 (C). **A)** ES1: The *left plot* represents eyes perfused at 35°C, whereas the *right plot* represents eyes perfused at 22°C (uncorrected value of *C_r_*). The *central plot* represents the value of *C_r_* for eyes perfused at 22°C after viscosity correction. The cello plots show all measured values of *C_r_* (data points) with the *error bars* representing the corresponding 95% CI on each measurement. The light shaded region shows the log-normal distribution that best fits the data. The central *white line* represents the geometric mean, and the outer white lines represent the range that encompasses 95% of the data. The central dark shaded region represents the 95% CI of the mean. (**B)** ES2: eyes perfused with metabolic inhibitors versus isosmotic vehicle (control). Both eyes are perfused at 35°C. Symbols are as defined in panel A. (**C)** ES3: The *left plot* represents eyes perfused at 35°C, whereas the *right plot* represents eyes perfused at 22°C (uncorrected value of *C_r_*), all eyes receiving with metabolic inhibitors. The *central plot* represents the value of *C_r_* for eyes perfused at 22°C after viscosity correction. Symbols are as defined in panel A.

**Figure 3.**
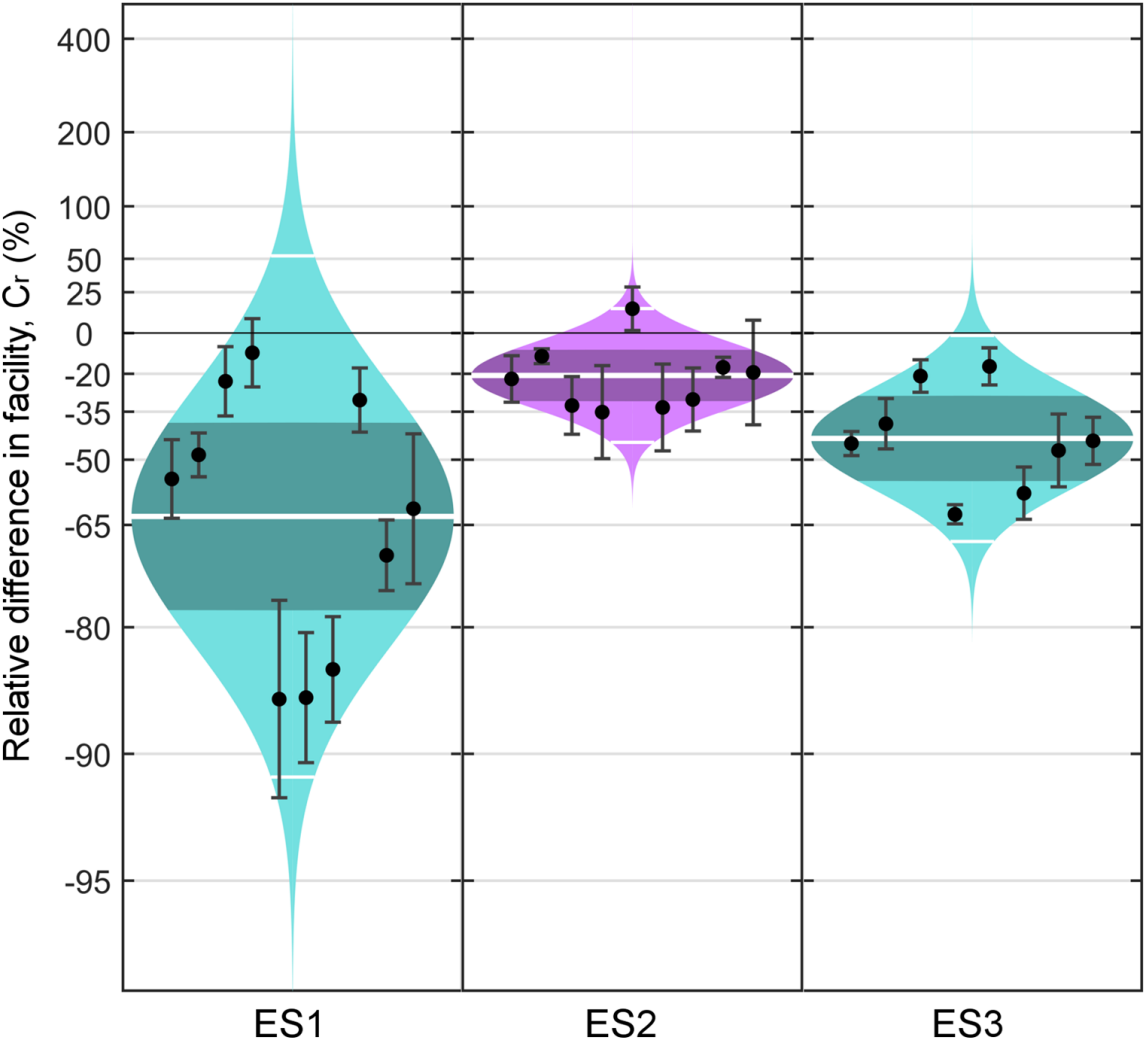
Cello plots showing the relative difference in outflow facility (*C_r_*) between contralateral eyes. *Left panel,* Outflow facility of eyes perfused at 22°C relative to 35°C with viscosity correction (ES1), all eyes perfused with vehicle. *Central panel,* Outflow facility of eyes perfused with metabolic inhibitors relative to isosmotic controls, all eyes perfused at 35°C (ES2). *Right panel,* Outflow facility of eyes perfused at 22°C relative to 35°C with viscosity correction, all eyes perfused with metabolic inhibitors (ES3). The symbols are as described in Figure 2.

ES1 examined the effect of temperature reduction on outflow facility (Fig. 2A). In eyes perfused at 35°C, *C_r_* was 4.8 [3.7, 6.3] nL/min/mm Hg (geometric mean [95% CI]). In eyes perfused at 22°C, *C_r_* before viscosity correction was 1.3 [0.9, 2.0] nL/min/mm Hg, but *C_r_* increased to 1.8 [1.2, 2.7] nL/min/mm Hg after viscosity correction. Between contralateral pairs, reducing temperature from 35°C to 22°C decreased *C_r_* by 63% [38%, 78%] after viscosity correction (*P* = 0.002, *n* = 10 pairs, Fig. 3 left panel).

ES2 examined the effect of metabolic inhibitors on outflow facility at physiological temperature (35°C; Fig. 2B). In eyes perfused with isosmotic vehicle, *C_r_* was 4.8 [2.4, 9.9] nL/min/mm Hg. In eyes perfused with metabolic inhibitors, *C_r_* was 3.8 [1.8, 7.8] nL/min/mm Hg. Between contralateral pairs, metabolic inhibitors reduced *C_r_* by 21% [9%, 31%] (*P* = 0.006, *n* = 9 pairs, Fig. 3, central panel). Data from one of the 10 pairs perfused was identified as an outlier and was not included in the statistical analysis.

ES3 examined the effect of temperature reduction in the presence of metabolic inhibitors (Fig. 2C). In eyes perfused at 35°C with metabolic inhibitors, *C_r_* was 7.2 [6.0, 8.6] nL/min/mm Hg. In eyes perfused at 22°C with metabolic inhibitors, *C_r_* before viscosity correction was 3.1 [2.6, 3.6] nL/min/mm Hg, which increased to 4.1 [3.4, 4.8] nL/min/mm Hg after viscosity correction. Between contralateral pairs both receiving metabolic inhibitors, reducing temperature from 35°C to 22°C decreased *C_r_* by 44% [56%, 29%] (*P* < 0.001, *n* = 8 pairs, Fig. 3 right panel). Two eyes of the 10 pairs perfused did not achieve the stability criteria described above, and data from both pairs were not included in the statistical analysis.

We also examined whether there was a relationship between the relative decrease in outflow facility and the baseline outflow facility, defined as the log-transformed value of *C_r_* in the control eye. For eyes treated with metabolic poisons in ES2, there was no detectible relationship between the relative change in facility and the value of ln *C_r_* in the control eye (*P* = 0.83, *n* = 9). However, for eyes experiencing temperature reduction in ES1 and ES3, there was a significant relationship, with a larger relative decrease in facility observed in eyes with a larger value of ln *C_r_* in the control eye (*P* = 0.013, η^2^ = 0.31, *n* = 18; ANCOVA).

### Effect of Temperature and Metabolic Inhibitors on ATP Levels in the Anterior Segment

**Figure 4.**
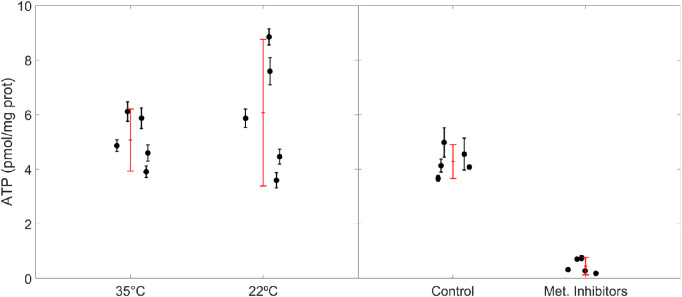
ATP levels in anterior segments of mouse eyes treated with temperature reduction (*left panel*) or metabolic inhibitors (*right panel*). Each data point represents the normalized ATP level (pmol ATP per mg of protein) obtained from one anterior segment with three technical replicates (mean ± SD). *Red symbols* represent the overall mean and 95% CI.

We measured how ATP levels were affected by temperature reduction and metabolic inhibitors (Fig. 4). In anterior segments at 35°C, the ATP level was 5.1 [3.9, 6.2] pmol/mg protein (mean [95% CI]), whereas in anterior segments at 22°C, the ATP level was 6.1 [3.4, 8.8] pmol/mg protein. These differences were not statistically significant between contralateral pairs (*P* = 0.36, *n* = 5 pairs). In anterior segments treated with metabolic inhibitors, the ATP level was 0.4 [0.1, 0.8] pmol/mg protein, whereas for eyes treated with isosmotic vehicle the ATP level was 4.3 [3.7, 4.9] pmol/mg protein. Between contralateral pairs, metabolic inhibitors reduced ATP levels by 90% [83%, 97%] (*P* << 0.001, *n* = 5 pairs).

## Discussion

Our results demonstrate that outflow facility in mice is sensitive to metabolic inhibitors and exquisitely sensitive to temperature. Reducing temperature from 35°C to 22°C reduced outflow facility by 63% on average in ES1, whereas metabolic inhibitors reduced outflow facility by 21% in ES2. We initially surmised that temperature reduction should effectively inhibit most enzymatic processes involved in metabolism, and thus we reasoned that temperature reduction should have a similar effect as metabolic inhibitors on outflow facility. However, to our surprise, we found that the effect of temperature reduction on outflow facility was threefold greater than metabolic inhibitors alone. Consistent with this notion, when controlling for the presence of metabolic inhibitors in ES3, temperature reduction from 35°C to 22°C still reduced outflow facility by 44%.

Intriguingly, the magnitude of these effects appeared to be additive. Specifically, the 63% facility decrease in response to temperature reduction was roughly equal to the sum of the effect of metabolic inhibitors alone (21%) and the effect of temperature reduction in the presence of metabolic inhibitors (44%). This suggests that temperature reduction suppresses both *metabolism-dependent* and *metabolism-independent* mechanisms to affect outflow facility. The metabolism-dependent mechanisms appear to account for roughly one fifth of normal outflow function in normal healthy adult mice, whereas the metabolism-independent and temperature-dependent mechanisms appear to account for just under one half of normal outflow function.

Before proceeding, it is worth benchmarking the effects of temperature reduction and metabolic inhibitors against other compounds that are known to reduce outflow facility in mice. Sphingosine 1-phosphate (S1P) decreases outflow facility by roughly 39% in enucleated mouse eyes,^12^ approximating the response observed in human eyes.^13^ S1P is thought to act by affecting cell contractility and stiffness via phosphorylation of myosin light chain.^39^ Dexamethasone (DEX) decreases outflow facility by approximately 52% in mice after three to four weeks of exposure, with effects attributed to increased extracellular matrix and basement membrane deposition underlying the inner wall of SC^40^ and increased TM stiffness.^41^ Blockade of vascular endothelial growth factor (VEGF) receptor 2 (VEGFR-2) with the Ki8751 or VEGF-A_165_b decreases outflow facility by 34% and 44%, respectively.^22^ Martentoxin (MarTX), which blocks the large-conductance potassium ion channels K_Ca_1.1 (or BK, maxi-K) that contain the β_4_ subunit, decreases outflow facility by roughly 35%, whereas iberiotoxin, which blocks K_Ca_1.1 channels that lack β_4_, decreases outflow facility by roughly 16%.^42^ Thus the effect of metabolic inhibitors on outflow facility is somewhat smaller than previously reported effects of S1P, DEX, MarTX, and VEGFR-2 antagonists. However, temperature reduction has the largest effect of any previously reported compound or treatment that has been shown to decrease outflow facility, at least in mice. Furthermore, it may be important that eyes with a higher baseline outflow facility exhibited a larger facility decrease in response to temperature reduction, suggesting that temperature-dependent mechanisms may contribute to differences in baseline outflow facility between individuals. Future studies should thus investigate how temperature reduction affects outflow function because this mechanism appears to have a critical role in outflow function.

### Comparison Against Prior Studies: Effects of Temperature

Our temperature studies in ES1 contrast with classic studies by VanBuskirk and Grant^2^ and Pollack et al*.*^43^ who did not find any significant change in outflow facility in response to temperature reduction in human and rabbit eyes respectively. Our results, however, are consistent with prior studies in mice from our own laboratory,^25^ as well as with data reported by Suzuki and Anderson^26^ in enucleated calf eyes.

VanBuskirk and Grant^2^ examined the effect of temperature reduction on outflow facility in 29 ostensibly normal enucleated human eyes. After correcting for changes in viscosity, they reported that “the reduction in temperature appeared to have little if any functional influence on the aqueous outflow itself.” Pollack et al*.*^43^ reported similar findings in live rabbits. Perhaps the outflow system in mice is more sensitive to temperature than other species. Alternatively, the effects of prolonged enucleation or postmortem time, which were less than six hours and <48 hours, respectively, in the human study,^2^ or advanced age of the donors could have suppressed the effects of temperature. In our study, young adult mouse eyes were perfused within 30 minutes of death, which may have better preserved the physiological function of the outflow pathway, but this does not explain the lack of the temperature effect observed in rabbits.^43^

Boussommier-Calleja et al*.*^25^ measured a 44% reduction in outflow facility in enucleated mouse eyes perfused at 20°C relative to 35°C after viscosity-correction. The mice in that study were of a similar genetic background and age as in our current study, but the perfusion in that study was done using an older pump-based perfusion system,^44^^,^^45^ rather than the reservoir-based iPerfusion system,^33^ which we believe to be more accurate. Additionally, because the contralateral eyes of that study^25^ were perfused consecutively, the postmortem time ranged up to three hours, with eyes stored at 4°C in PBS after enucleation, which occurred within 15 minutes of death. It is thus possible that the longer postmortem times or less-accurate techniques used in our previous study^25^ may have reduced the apparent effect of temperature on outflow facility relative to our current work.

Suzuki and Anderson^26^ examined how temperature reduction influenced the action of metabolic inhibitors on outflow facility in enucleated calf eyes. Although the effect of temperature alone was not the main focus of their study, their data suggest a 47% reduction in the measured outflow facility at 22°C relative to 36°C in the absence of metabolic inhibitors. There was no mention of whether these differences achieved statistical significance or whether the facility values were corrected for changes in viscosity. If viscosity changes were not accounted for in their reported data, then applying the correction yields a temperature-dependent facility decrease of 28%. The postmortem time was not reported, but as the eyes were obtained from a local abattoir, it was likely at least a few hours, with the eyes kept cold until use.

The discrepancy between these studies,^2^^,^^25^^,^^26^^,^^43^ requires further investigation to determine whether, as previously suggested, postmortem time,^46^^,^^47^ in addition to age, species, or methodologic differences (e.g., enucleation) may explain the temperature sensitivity to outflow observed in mouse (and possibly calf) eyes versus in human and rabbit eyes. If species differences are found to be responsible and the ex vivo mouse eye is indeed unique in regard to temperature-sensitive outflow, then this would raise questions about the validity of the ex vivo mouse eyes (and potentially mouse models in general) for investigating outflow physiology, particularly if such temperature sensitivity is not observed in primates.

### Comparison Against Prior Studies: Effects of Metabolic Inhibitors

**Figure 5.**
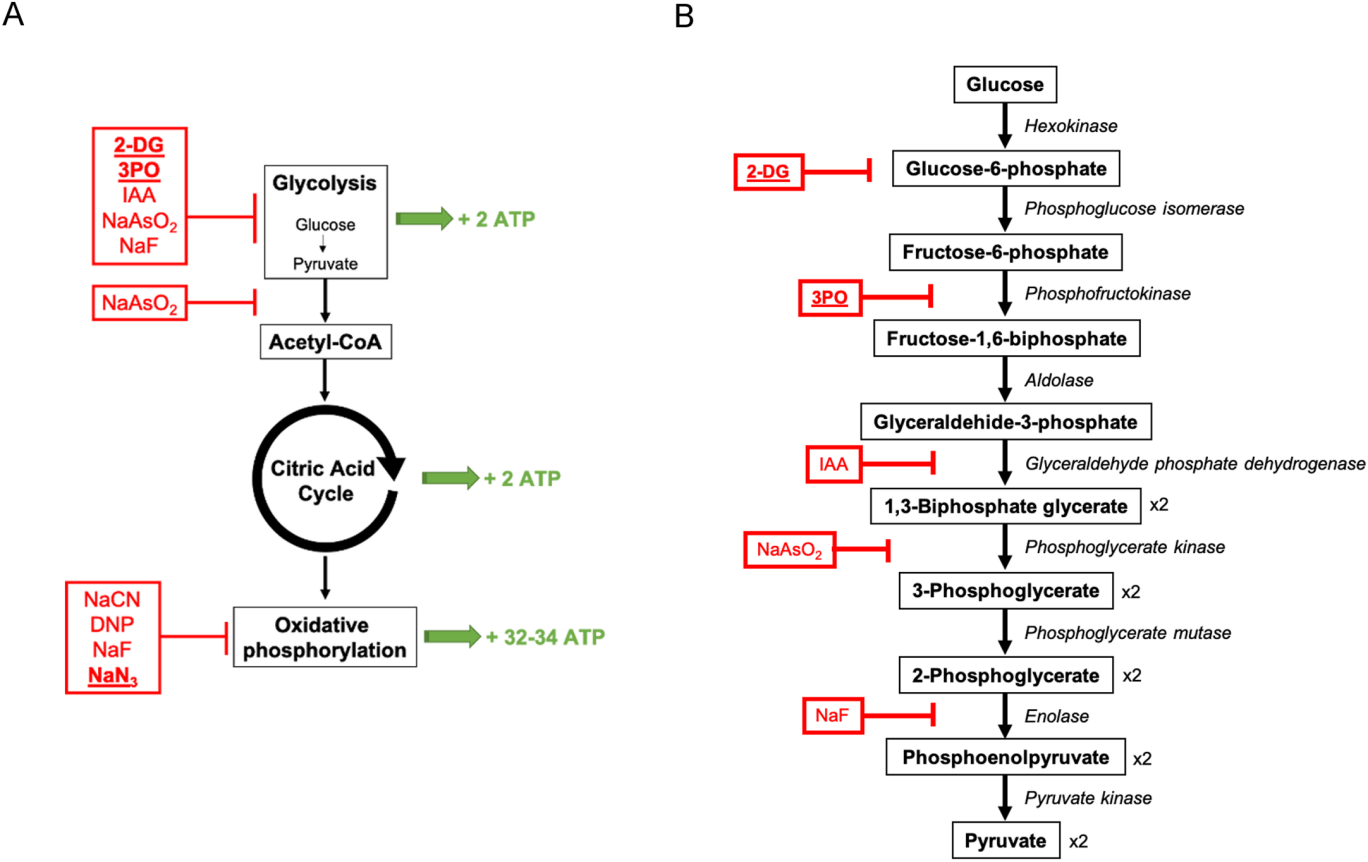
**(A)** A summary of the main metabolic pathway for ATP production and where metabolic inhibitors affect this pathway. Inhibitors that are underlined are those used in this study, whereas those that are not underlined were used by Bárány.^1^ The net ATP production per molecule of glucose is shown in green. (**B)** Schematic representation of glycolysis, with metabolic inhibitors shown in red. 2-DG: 2-Deoxy-D-glucose; 3PO: (2E)-3-(3-Pyridinyl)-1-(4-pyridinyl)-2-propen-1-one; IAA: iodoacetic acid; NaAsO_2_: sodium arsenite; NaF: sodium fluoride; NaCN: sodium cyanide; NaN_3_: sodium azide; DNP: 2-4-Dinitrophenol.

Bárány^1^ studied the effect of several metabolic inhibitors on outflow facility in enucleated eyes from cattle. Figure 5 summarizes the inhibitors used by Bárány, which target glycolysis (iodoacetic acid [IAA]; sodium arsenite [NaAsO_2_]; sodium fluoride [NaF]), production of acetyl-coenzyme A (NaAsO_2_), and oxidative phosphorylation (NaF; NaN_3_; sodium cyanide [NaCN]; 2-4-dinitrophenol [DNP]). None of these compounds perfused on their own were found to have any effect on outflow, except for IAA, which increased outflow facility, likely via its sulfhydryl effects rather than via its metabolic effects.^26^^,^^48^^,^^49^

In Bárány's study^1^ each inhibitor was examined in only three pairs of eyes, and thus it is possible that any effects could have been simply missed due to small sample sizes. Furthermore, Bárány's perfusions were performed at room temperature,^1^ where effects of metabolic inhibitors may be suppressed. For example, Suzuki and Anderson^26^ showed that NaF had a negligible effect on outflow facility at room temperature, but NaF decreased facility by as much as 50% (average of 30%) at physiological temperature. This is consistent with our finding that temperature reduction has a larger effect on outflow than metabolic inhibitors.

Suzuki and Anderson^26^ examined the effect of DNP, 2-DG, IAA, iodoacetamide (IA) and potassium cyanide (KCN) on outflow facility in enucleated calf eyes. IAA and IA increased facility, consistent with findings by Bárány,^1^ but with the exception of NaF, no other inhibitor was found to have any effect on outflow facility. Importantly, NaF is an inhibitor of both glycolysis (via enolase^50^) and oxidative phosphorylation^51^^,^^52^ (Fig. 5), and, hence, NaF is similar to our inhibitor cocktail by targeting multiple metabolic pathways simultaneously. The metabolism of the trabecular meshwork is thought to be largely anaerobic and driven by glycolysis.^53^ However, inhibition of one metabolic pathway may lead to compensation by other pathways that may make use of alternative energy sources. Thus a single inhibitor that targets multiple pathways, such as NaF, or an inhibitor cocktail may be more effective at examining the role of active cellular metabolism in outflow function.

### Effects on ATP Levels

Our data show that simultaneous inhibition of glycolysis and oxidative phosphorylation reduces ATP levels by nearly 90% after two hours. These measurements provide biochemical validation that our inhibitory cocktail was functional within the mouse eye. These ATP measurements were made of the anterior corneoscleral shell, but we may assume with reasonable confidence that ATP levels in the TM would follow similar trends. Although we point out that measurements of ATP in the anterior segment as a whole may not be entirely reflective of changes within the TM and may be strongly influenced by more metabolically active tissues such as the cornea. For instance, the integrity of the cornea has been used as indicator of the health of the trabecular meshwork.^54^ Presumably, the metabolic inhibitors effectively suppressed ATP production without affecting consumption, such that ATP levels declined after exposure to the inhibitors. Because we only measured ATP levels after two hours of exposure, at a time point corresponding to the approximate end of the perfusion, we cannot comment on the time course of ATP depletion. Thus, it could have been possible that ATP levels were not yet fully exhausted at the earlier stages of the perfusion. If that were the case, however, we should expect the true effect of metabolic inhibitors on outflow facility to be even larger than the 21% effect measured in this study. Regardless, these results demonstrate that inhibiting glycolysis or oxidative phosphorylation coincides with a reduction in outflow facility, suggesting that metabolic production of ATP is necessary to maintain roughly one fifth of normal outflow function.

ATP levels were not significantly affected by temperature reduction. This is likely because temperature reduction, in contrast to the metabolic inhibitors, affects both the consumption and production of ATP. Both processes involve enzymatic activity, and all enzymes have an optimal temperature where the rate of reaction is greatest. As a general rule, a temperature change of 1° or 2°C may introduce 10% to 20% change in enzyme reaction rate.^55^ If the rates of production and consumption of ATP are decreased similarly by temperature reduction, then we would expect ATP levels to remain fairly constant, as we had observed. Otherwise, any increase in the consumption rate must have been offset by an increase in the production rate, which seems unlikely. It thereby seems reasonable to assume that temperature reduction decreases the rate of ATP consumption in the anterior segment and, by extension, the conventional outflow pathway.

Thus, despite ATP levels being maintained, the decrease in ATP consumption imposed by temperature reduction should have had a similar metabolic effect as ATP depletion, because both processes effectively inhibit the downstream effects of ATP. To our surprise, temperature reduction had a threefold larger effect on outflow facility than metabolic inhibitors. A portion of the total effect of temperature reduction can be attributed to suppression of metabolic activity, but even in the presence of metabolic inhibitors, temperature reduction decreased outflow facility by a further 44%. This reveals that temperature-reduction affects both metabolism (or ATP)-dependent mechanisms and ATP-independent mechanisms to impair outflow function. Understanding these separate mechanisms is important for understanding the basis for outflow resistance generation.

### Implications for Outflow Resistance Generation

The current consensus attributes the majority of outflow resistance generation to flow through extracellular matrix material within the juxtacanalicular connective tissue (JCT) immediately underling the inner wall endothelium of SC.^56^^,^^57^ Resistance generated via this mechanism should be almost entirely passive (ATP-independent) and temperature-insensitive (apart from the effects on aqueous humor viscosity). Our results are thus at odds with the view that extracellular matrix in the JCT is *solely* responsible for resistance generation. However, it is now fairly well accepted that outflow resistance generation arises because of a synergistic interaction between the inner wall and JCT.^58^ Regardless of the details of any particular synergistic mechanism (e.g., funnelling^45^^,^^59^), our results demonstrate that TM or SC cells must be directly involved in outflow resistance generation, at least in mice. In other words, flow through extracellular matrix cannot be the sole mechanism of outflow resistance generation.

TM and SC cells contribute to development and maintenance of the outflow pathway. It is thus not entirely surprising that inhibiting the metabolism of these cells would disrupt outflow function. What it is surprising, however, is the timescale of the observed effects. Our results reveal that inhibiting metabolism or reducing temperature has practically an immediate inhibitory effect on outflow function. Such a fast response is inconsistent with the slower timescale of extracellular matrix remodeling and turnover, which typically operates over several hours to days.^60^ This suggests that TM and SC cells may function over a fast timescale to modulate outflow resistance, with extracellular matrix providing a longer-lasting, more stable structure that provides the basis for resistance generation.^61^^–^^63^

There are ample means by which TM and SC cells may quickly modulate outflow resistance without requiring active transport of aqueous humor per se. Focal extracellular matrix remodeling by podosome or invadopodia-like structures^64^ occurs over the timescale of several minutes and would be inhibited by ATP-depletion or temperature reduction. Signaling pathways involving NO,^21^^,^^65^^–^^67^ VEGF,^22^ pigment epithelium-derived factor (PEDF),^23^ transforming growth factor–β^24^^,^^68^ and prostaglandins^20^^,^^69^^,^^70^ have all been shown to quickly modulate outflow resistance and require metabolic energy. Modulation of cell junctions,^71^^–^^76^ cytoskeletal contractility,^15^^,^^77^^–^^82^ cell remodelling,^83^ and even ATP-sensitive^42^^,^^84^ or temperature-dependent^85^^,^^86^ ion channels may contribute as well. ATP depletion or temperature reduction could also affect the mechanical properties of TM and SC cells, as in other cell types,^87^ to affect outflow resistance via cell or tissue stiffness or rheology.^58^^,^^88^^,^^89^ Future work should examine the basis for how inhibition of metabolism and temperature reduction in particular affects outflow resistance generation.

In conclusion, this study reveals that cells in the outflow pathway actively modulate outflow function over fast timescales through both metabolism-dependent and metabolism-independent mechanisms. Metabolism-dependent mechanisms account for roughly one fifth of total outflow function in normal healthy adult mice, whereas metabolism-independent cellular mechanisms account for just under one half of outflow function. This challenges the prevailing view that aqueous humor outflow is largely passive, at least in mice, and further emphasizes the need to understand the mechanisms by which TM and SC cells actively modulate outflow resistance generation. This study also reveals a yet unidentified mechanism that is exquisitely temperature-sensitive but occurs independently of ATP that contributes to roughly half of outflow function.

## References

[1] Bárány EH In vitro studies of the resistance to flow through the angle of the anterior chamber. *Acta Soc Med Ups*. 1954; 59: 260–276. 13158168

[2] VanBuskirk EM, Grant WM Influence of temperature and the question of involvement of cellular metabolism in aqueous outflow. *Am J Ophthalmol*. 1974; 77: 565–572. 4819458 10.1016/0002-9394(74)90472-3

[3] Johnstone MA CHAPTER 3 - Aqueous humor outflow system overview. In: Lieberman MF, Drake MV (eds), *Becker-Shaffer's diagnosis and therapy of the glaucomas (8th Edition)*. Edinburgh: Mosby; 2009: 25–46.

[4] Johnson M, Erickson K Mechanisms and routes of aqueous humor drainage. In: Albert DM, Jakobiec F.A. (ed), *Principles and Practice of Ophthalmology*. Saunders, Philadelphia: W.B Saunders Company; 2000: 2577–2595.

[5] Millar JC, Kaufman PL Aqueous humor: Secretion and dynamics. *Duane's foundations of clinical ophthalmology*. Philadelphia: Lippincott-Raven; 1995: 1–51.

[6] Toris CB, Tye G, Pattabiraman P Changes in Parameters of Aqueous Humor Dynamics Throughout Life. In: Guidoboni G, Harris A, Sacco R (eds), *Ocular Fluid Dynamics: Anatomy, Physiology, Imaging Techniques, and Mathematical Modeling*. Cham: Springer International Publishing; 2019: 161–190.

[7] Barton K, A. HR Pathogenesis of Glaucoma. In: Budenz DL (ed), *Medical Management of Glaucoma*: Springer Healthcare, Tarporley; 2013: 33–48.

[8] Goel M, Picciani RG, Lee RK, Bhattacharya SK Aqueous humor dynamics: a review. *Open Ophthalmol J*. 2010; 4: 52–59. 21293732 10.2174/1874364101004010052 PMC3032230

[9] Tamm ER The trabecular meshwork outflow pathways: Structural and functional aspects. *Exp Eye Res*. 2009; 88: 648–655. 19239914 10.1016/j.exer.2009.02.007

[10] Pizzirani S, Gong H Functional Anatomy of the Outflow Facilities. *Vet Clin North Am Small Anim Pract*. 2015; 45: 1101–1126. 26337760 10.1016/j.cvsm.2015.06.005 PMC4787989

[11] Andrew NH, Akkach S, Casson RJ A review of aqueous outflow resistance and its relevance to microinvasive glaucoma surgery. *Surv Ophthalmol*. 2019; 65: 18–31. 31425701 10.1016/j.survophthal.2019.08.002

[12] Boussommier-Calleja A, Bertrand J, Woodward DF, et al. Pharmacologic manipulation of conventional outflow facility in ex vivo mouse eyes. *Invest Ophthalmol Vis Sci*. 2012; 53: 5838–5845. 22807298 10.1167/iovs.12-9923 PMC3428113

[13] Stamer WD, Read AT, Sumida GM, Ethier CR Sphingosine-1-phosphate effects on the inner wall of Schlemm's canal and outflow facility in perfused human eyes. *Exp Eye Res*. 2009; 89: 980–988. 19715693 10.1016/j.exer.2009.08.008 PMC2794662

[14] Mettu PS, Deng PF, Misra UK, et al. Role of lysophospholipid growth factors in the modulation of aqueous humor outflow facility. *Invest Ophthalmol Vis Sci*. 2004; 45: 2263–2271. 15223804 10.1167/iovs.03-0960

[15] Rao PV, Deng PF, Kumar J, Epstein DL Modulation of aqueous humor outflow facility by the Rho kinase-specific inhibitor Y-27632. *Invest Ophthalmol Vis Sci*. 2001; 42: 1029–1037. 11274082

[16] Wang JW, Woodward DF, Stamer WD Differential effects of prostaglandin E2-sensitive receptors on contractility of human ocular cells that regulate conventional outflow. *Invest Ophthalmol Vis Sci*. 2013; 54: 4782–4790. 23766471 10.1167/iovs.13-12363 PMC3719448

[17] Wiederholt M Direct involvement of trabecular meshwork in the regulation of aqueous humor outflow. *Curr Opin Ophthalmol*. 1998; 9: 46–49. 10.1097/00055735-199804000-00009 10180513

[18] Hoffman EA, Perkumas KM, Highstrom LM, Stamer WD Regulation of myocilin-associated exosome release from human trabecular meshwork cells. *Invest Ophthalmol Vis Sci*. 2009; 50: 1313–1318. 18952916 10.1167/iovs.08-2326 PMC2671074

[19] Stamer WD, Hoffman EA, Luther JM, Hachey DL, Schey KL Protein profile of exosomes from trabecular meshwork cells. *J Proteomics*. 2011; 74: 796–804. 21362503 10.1016/j.jprot.2011.02.024 PMC3085584

[20] Millard LH, Woodward DF, Stamer WD The role of the prostaglandin EP4 receptor in the regulation of human outflow facility. *Invest Ophthalmol Vis Sci*. 2011; 52: 3506–3513. 21245402 10.1167/iovs.10-6510

[21] Chang JY, Stamer WD, Bertrand J, et al. Role of nitric oxide in murine conventional outflow physiology. *Am J Physiol Cell Physiol*. 2015; 309: C205–214. 26040898 10.1152/ajpcell.00347.2014 PMC4537932

[22] Reina-Torres E, Wen JC, Liu KC, et al. VEGF as a paracrine regulator of conventional outflow facility. *Invest Ophthalmol Vis Sci*. 2017; 58: 1899–1908. 28358962 10.1167/iovs.16-20779 PMC5374885

[23] Rogers ME, Navarro ID, Perkumas KM, et al. Pigment epithelium-derived factor decreases outflow facility. *Invest Ophthalmol Vis Sci*. 2013; 54: 6655–6661. 24030458 10.1167/iovs.13-12766 PMC3796938

[24] Fuchshofer R, Tamm ER The role of TGF-beta in the pathogenesis of primary open-angle glaucoma. *Cell Tissue Res*. 2012; 347: 279–290. 22101332 10.1007/s00441-011-1274-7

[25] Boussommier-Calleja A, Li G, Wilson A, et al. Physical factors affecting outflow facility measurements in mice. *Invest Ophthalmol Vis Sci*. 2015; 56: 8331–8339. 26720486 10.1167/iovs.15-17106 PMC4699419

[26] Suzuki R, Anderson PJ A temperature dependent action of fluoride on aqueous outflow facility of the calf eye. *Curr Eye Res*. 1993; 12: 1–7. 8436006 10.3109/02713689308999489

[27] Woodward GE, Hudson MT. The effect of 2-desoxy-D-glucose on glycolysis and respiration of tumor and normal tissues. *Cancer Res*. 1954; 14: 599–605. 13199805

[28] Wick AN, Drury DR, Nakada HI, Wolfe JB Localization of the primary metabolic block produced by 2-deoxyglucose. *J Biol Chem*. 1957; 224: 963–969. 13405925

[29] Clem B, Telang S, Clem A, et al. Small-molecule inhibition of 6-phosphofructo-2-kinase activity suppresses glycolytic flux and tumor growth. *Mol Cancer Ther*. 2008; 7: 110–120. 18202014 10.1158/1535-7163.MCT-07-0482

[30] Xintaropoulou C, Ward C, Wise A, et al. A comparative analysis of inhibitors of the glycolysis pathway in breast and ovarian cancer cell line models. *Oncotarget*. 2015; 6: 25677–25695. 26259240 10.18632/oncotarget.4499 PMC4694858

[31] Keilin D The action of sodium azide on cellular respiration and on some catalytic oxidation reactions. *Proc R Soc London Series B Biol Sci*. 1936; 121: 165–173.

[32] Ishikawa T, Zhu BL, Maeda H Effect of sodium azide on the metabolic activity of cultured fetal cells. *Toxicol Ind Health*. 2006; 22: 337–341. 17120532 10.1177/0748233706071737

[33] Sherwood JM, Reina-Torres E, Bertrand JA, Rowe B, Overby DR Measurement of outflow facility using iPerfusion. *PLoS One*. 2016; 11: e0150694. 26949939 10.1371/journal.pone.0150694 PMC4780770

[34] Madekurozwa M, Reina-Torres E, Overby DR, Sherwood JM Direct measurement of pressure-independent aqueous humour flow using iPerfusion. *Exp Eye Res*. 2017; 162: 129–138. 28720436 10.1016/j.exer.2017.07.008 PMC5587799

[35] Huber ML, Perkins RA, Laesecke A, et al. New international formulation for the viscosity of H2O. *J Phys Chem Ref Data*. 2009; 38: 101–125.

[36] Sunyer R, Trepat X, Fredberg JJ, Farré R, Navajas D The temperature dependence of cell mechanics measured by atomic force microscopy. *Phys Biol*. 2009; 6: 025009. 19571363 10.1088/1478-3975/6/2/025009 PMC3932184

[37] Burdyga TV, Wray S On the mechanisms whereby temperature affects excitation-contraction coupling in smooth muscle. *J Gen Physiol*. 2002; 119: 93–104. 11773241 10.1085/jgp.119.1.93 PMC2233859

[38] Nasu T Effects of cooling on smooth muscle contraction. *Comp Biochem Physiol A Comp Physiol*. 1990; 95: 201–207. 1968816 10.1016/0300-9629(90)90199-3

[39] Sumida GM, Stamer WD Sphingosine-1-phosphate enhancement of cortical actomyosin organization in cultured human Schlemm's canal endothelial cell monolayers. *Invest Ophthalmol Vis Sci*. 2010; 51: 6633–6638. 20592229 10.1167/iovs.10-5391 PMC3055773

[40] Overby DR, Bertrand J, Tektas OY, et al. Ultrastructural changes associated with dexamethasone-induced ocular hypertension in mice. *Invest Ophthalmol Vis Sci*. 2014; 55: 4922–4933. 25028360 10.1167/iovs.14-14429 PMC4126794

[41] Li G, Lee C, Agrahari V, et al. In vivo measurement of trabecular meshwork stiffness in a corticosteroid-induced ocular hypertensive mouse model. *Proc Natl Acad Sci USA*. 2019; 116: 1714–1722. 30651311 10.1073/pnas.1814889116 PMC6358695

[42] Bertrand JA, Schicht M, Stamer WD, et al. The beta4-subunit of the Large-Conductance Potassium Ion Channel KCa1.1 regulates outflow facility in mice. *Invest Ophthalmol Vis Sci*. 2020; 61: 41. 10.1167/iovs.61.3.41 PMC7401454 32203982

[43] Pollack IP, Becker B, Constant MA The effect of hypothermia on aqueous humor dynamics. I. Intraocular pressure and outflow facility of the rabbit eye. *Am J Ophthalmol*. 1960; 49: 1126–1134. 14433835 10.1016/0002-9394(60)91624-x

[44] Lei Y, Overby DR, Boussommier-Calleja A, Stamer WD, Ethier CR Outflow physiology of the mouse eye: pressure dependence and washout. *Invest Ophthalmol Vis Sci*. 2011; 52: 1865–1871. 21169533 10.1167/iovs.10-6019 PMC3101677

[45] Overby DR, Gong H, Qiu G, Freddo TF, Johnson M The mechanism of increasing outflow facility during washout in the bovine eye. *Invest Ophthalmol Vis Sci*. 2002; 43: 3455–3464. 12407156

[46] Gual A, Llobet A, Gilabert R, et al. Effects of time of storage, albumin, and osmolality changes on outflow facility (C) of bovine anterior segment in vitro. *Invest Ophthalmol Vis Sci*. 1997; 38: 2165–2171. 9331281

[47] Acott TS, Kingsley PD, Samples JR, Vanbuskirk EM Human trabecular meshwork organ-culture - Morphology and glycosaminoglycan synthesis. *Invest Ophthalmol Vis Sci*. 1988; 29: 90–100. 3335436

[48] Epstein DL, Hashimoto JM, Anderson PJ, Grant WM Effect of iodoacetamide perfusion on outflow facility and metabolism of the trabecular meshwork. *Invest Ophthalmol Vis Sci*. 1981; 20: 625–631. 6783588

[49] Lindenmayer JM, Kahn MG, Hertzmark E, Epstein DL Morphology and function of the aqueous outflow system in monkey eyes perfused with sulfhydryl reagents. *Invest Ophthalmol Vis Sci*. 1983; 24: 710–717. 6853097

[50] Qin J, Chai G, Brewer JM, Lovelace LL, Lebioda L Fluoride inhibition of enolase: crystal structure and thermodynamics. *Biochemistry*. 2006; 45: 793–800. 16411755 10.1021/bi051558s PMC2566932

[51] Gutowska I, Baranowska-Bosiacka I, Baskiewicz M, et al. Fluoride as a pro-inflammatory factor and inhibitor of ATP bioavailability in differentiated human THP1 monocytic cells. *Toxicol Lett*. 2010; 196: 74–79. 20399260 10.1016/j.toxlet.2010.03.1167

[52] Lovelace CJ, Miller GW In vitro effects of fluoride on tricarboxylic acid cycle dehydrogenases and oxidative phosphorylation. I. *J Histochem Cytochem*. 1967; 15: 195–201. 4291791 10.1177/15.4.195

[53] Anderson PJ, Wang J, Epstein DL Metabolism of calf trabecular (reticular) meshwork. *Invest Ophthalmol Vis Sci*. 1980; 19: 13–20. 7350129

[54] Wan Z, Brigatti L, Ranger-Moore J, Ethier CR, Stamer WD Rate of change in central corneal thickness: a viability indicator for conventional drainage tissues in organ culture. *Exp Eye Res*. 2006; 82: 1086–1093. 16466713 10.1016/j.exer.2005.10.027

[55] Worthington Biochemical Corporation, Worthington Diagnostics. *Manual of clinical enzyme measurements*: Worthington, OH: Worthington Diagnostics; 1972.

[56] Mäepea O, Bill A Pressures in the juxtacanalicular tissue and Schlemm's canal in monkeys. *Exp Eye Res*. 1992; 54: 879–883. 1521580 10.1016/0014-4835(92)90151-h

[57] Lütjen-Drecoll E Structural factors influencing outflow facility and its changeability under drugs. A study in Macaca arctoides. *Invest Ophthalmol Vis Sci*. 1973; 12: 280–294. 4144361

[58] Overby DR, Stamer WD, Johnson M The changing paradigm of outflow resistance generation: towards synergistic models of the JCT and inner wall endothelium. *Exp Eye Res*. 2009; 88: 656–670. 19103197 10.1016/j.exer.2008.11.033 PMC2744486

[59] Johnson M, Shapiro A, Ethier CR, Kamm RD Modulation of outflow resistance by the pores of the inner wall endothelium. *Invest Ophthalmol Vis Sci*. 1992; 33: 1670–1675. 1559767

[60] Vittal V, Rose A, Gregory KE, Kelley MJ, Acott TS Changes in gene expression by trabecular meshwork cells in response to mechanical stretching. *Invest Ophthalmol Vis Sci*. 2005; 46: 2857–2868. 16043860 10.1167/iovs.05-0075

[61] Keller KE, Aga M, Bradley JM, Kelley MJ, Acott TS Extracellular matrix turnover and outflow resistance. *Exp Eye Res*. 2009; 88: 676–682. 19087875 10.1016/j.exer.2008.11.023 PMC2700052

[62] Keller KE, Bradley JM, Vranka JA, Acott TS Segmental versican expression in the trabecular meshwork and involvement in outflow facility. *Invest Ophthalmol Vis Sci*. 2011; 52: 5049–5057. 21596823 10.1167/iovs.10-6948 PMC3176074

[63] Acott TS, Kelley MJ Extracellular matrix in the trabecular meshwork. *Exp Eye Res*. 2008; 86: 543–561. 18313051 10.1016/j.exer.2008.01.013 PMC2376254

[64] Aga M, Bradley JM, Keller KE, Kelley MJ, Acott TS Specialized podosome- or invadopodia-like structures (PILS) for focal trabecular meshwork extracellular matrix turnover. *Invest Ophthalmol Vis Sci*. 2008; 49: 5353–5365. 18641286 10.1167/iovs.07-1666 PMC2683617

[65] Ashpole NE, Overby DR, Ethier CR, Stamer WD Shear stress-triggered nitric oxide release from Schlemm's canal cells. *Invest Ophthalmol Vis Sci*. 2014; 55: 8067–8076. 25395486 10.1167/iovs.14-14722 PMC4266075

[66] Doganay S, Evereklioglu C, Turkoz Y, Er H Decreased nitric oxide production in primary open-angle glaucoma. *Eur J Ophthalmol*. 2002; 12: 44–48. 11936443 10.1177/112067210201200109

[67] Stamer WD, Lei Y, Boussommier-Calleja A, Overby DR, Ethier CR eNOS, a pressure-dependent regulator of intraocular pressure. *Invest Ophthalmol Vis Sci*. 2011; 52: 9438–9444. 22039240 10.1167/iovs.11-7839 PMC3293415

[68] Gottanka J, Chan D, Eichhorn M, Lutjen-Drecoll E, Ethier CR Effects of TGF-beta2 in perfused human eyes. *Invest Ophthalmol Vis Sci*. 2004; 45: 153–158. 14691167 10.1167/iovs.03-0796

[69] Bahler CK, Howell KG, Hann CR, Fautsch MP, Johnson DH Prostaglandins increase trabecular meshwork outflow facility in cultured human anterior segments. *Am J Ophthalmol*. 2008; 145: 114–119. 17988642 10.1016/j.ajo.2007.09.001 PMC2745953

[70] Winkler NS, Fautsch MP Effects of prostaglandin analogues on aqueous humor outflow pathways. *J Ocul Pharmacol Ther*. 2014; 30: 102–109. 24359106 10.1089/jop.2013.0179 PMC3991965

[71] Heimark RL, Kaochar S, Stamer WD Human Schlemm's canal cells express the endothelial adherens proteins, VE-cadherin and PECAM-1. *Curr Eye Res*. 2002; 25: 299–308. 12658549 10.1076/ceyr.25.5.299.13495

[72] Tam LC, Reina-Torres E, Sherwood JM, et al. Enhancement of outflow facility in the murine eye by targeting selected tight-junctions of Schlemm's canal endothelia. *Sci Rep*. 2017; 7: 40717. 28091584 10.1038/srep40717 PMC5238500

[73] Pattabiraman PP, Epstein DL, Rao PV Regulation of adherens junctions in trabecular meshwork cells by Rac GTPase and their influence on intraocular pressure. *J Ocul Biol*. 2013; 1: 0002. 24932460 10.13188/2334-2838.1000002 PMC4057051

[74] Cantelmo AR, Conradi LC, Brajic A, et al. Inhibition of the Glycolytic Activator PFKFB3 in Endothelium Induces Tumor Vessel Normalization, Impairs Metastasis, and Improves Chemotherapy. *Cancer Cell*. 2016; 30: 968–985. 27866851 10.1016/j.ccell.2016.10.006 PMC5675554

[75] Denker BM, Nigam SK Molecular structure and assembly of the tight junction. *Am J Physiol*. 1998; 274: F1–F9. 9458817 10.1152/ajprenal.1998.274.1.F1

[76] Tsukamoto T, Nigam SK Tight junction proteins form large complexes and associate with the cytoskeleton in an ATP depletion model for reversible junction assembly. *J Biol Chem*. 1997; 272: 16133–16139. 9195909 10.1074/jbc.272.26.16133

[77] Pattabiraman PP, Inoue T, Rao PV Elevated intraocular pressure induces Rho GTPase mediated contractile signaling in the trabecular meshwork. *Exp Eye Res*. 2015; 136: 29–33. 25956210 10.1016/j.exer.2015.05.001 PMC4466129

[78] Kameda T, Inoue T, Inatani M, et al. The effect of Rho-associated protein kinase inhibitor on monkey Schlemm's canal endothelial cells. *Invest Ophthalmol Vis Sci*. 2012; 53: 3092–3103. 22491412 10.1167/iovs.11-8018

[79] Lu Z, Overby DR, Scott PA, Freddo TF, Gong H The mechanism of increasing outflow facility by rho-kinase inhibition with Y-27632 in bovine eyes. *Exp Eye Res*. 2008; 86: 271–281. 18155193 10.1016/j.exer.2007.10.018 PMC2441864

[80] Epstein DL, Rowlette LL, Roberts BC Acto-myosin drug effects and aqueous outflow function. *Invest Ophthalmol Vis Sci*. 1999; 40: 74–81. 9888429

[81] Ethier CR, Read AT, Chan DW Effects of latrunculin-B on outflow facility and trabecular meshwork structure in human eyes. *Invest Ophthalmol Vis Sci*. 2006; 47: 1991–1998. 16639007 10.1167/iovs.05-0327

[82] Li A, Leung CT, Peterson-Yantorno K, Stamer WD, Civan MM Cytoskeletal dependence of adenosine triphosphate release by human trabecular meshwork cells. *Invest Ophthalmol Vis Sci*. 2011; 52: 7996–8005. 21896846 10.1167/iovs.11-8170 PMC3220413

[83] Junk AK, Goel M, Mundorf T, Rockwood EJ, Bhattacharya SK Decreased carbohydrate metabolism enzyme activities in the glaucomatous trabecular meshwork. *Mol Vis*. 2010; 16: 1286–1291. 20664702 PMC2904041

[84] Chowdhury UR, Bahler CK, Hann CR, et al. ATP-sensitive potassium (KATP) channel activation decreases intraocular pressure in the anterior chamber of the eye. *Invest Ophthalmol Vis Sci*. 2011; 52: 6435–6442. 21743021 10.1167/iovs.11-7523 PMC3176023

[85] Yarishkin O, Phuong TTT, Bretz CA, et al. TREK-1 channels regulate pressure sensitivity and calcium signaling in trabecular meshwork cells. *J Gen Physiol*. 2018; 150: 1660–1675. 30446509 10.1085/jgp.201812179 PMC6279358

[86] Ryskamp DA, Frye AM, Phuong TT, et al. TRPV4 regulates calcium homeostasis, cytoskeletal remodeling, conventional outflow and intraocular pressure in the mammalian eye. *Sci Rep*. 2016; 6: 30583. 27510430 10.1038/srep30583 PMC4980693

[87] Fabry B, Maksym GN, Butler JP, et al. Time scale and other invariants of integrative mechanical behavior in living cells. *Phys Rev E Stat Nonlin Soft Matter Phys*. 2003; 68: 041914. 14682980 10.1103/PhysRevE.68.041914

[88] Vahabikashi A, Gelman A, Dong B, et al. Increased stiffness and flow resistance of the inner wall of Schlemm's canal in glaucomatous human eyes. *Proc Natl Acad Sci USA*. 2019; 116: 26555–26563. 10.1073/pnas.1911837116 PMC6936716 31806762

[89] Vranka JA, Staverosky JA, Reddy AP, et al. Biomechanical rigidity and quantitative proteomics analysis of segmental regions of the trabecular meshwork at physiologic and elevated pressures. *Invest Ophthalmol Vis Sci*. 2018; 59: 246–259. 29340639 10.1167/iovs.17-22759 PMC5770183

